# Material Behavior and Computational Validation of Deep CO_2_ Closed-Loop Geothermal Systems in Carbonate Reservoirs

**DOI:** 10.3390/ma18225144

**Published:** 2025-11-12

**Authors:** Xinghui Wu, Peng Li, Meifeng Cai, Tingting Jiang, Bolin Mu, Wanlei Su, Min Wang, Chunxiao Li

**Affiliations:** 1 School of City and Architecture Engineering, Zaozhuang University, Zaozhuang 277160, China; 2 Beijing Key Laboratory of Urban Underground Space Engineering, University of Science and Technology Beijing, Beijing 100083, China; 3 School of Resources Environment and Safety Engineering, University of South China, Hengyang 421001, China

**Keywords:** finite element analysis, material behavior, computational validation, CO_2_ closed-loop system, multi-objective optimization

## Abstract

Closed-loop geothermal systems (CLGSs) avoid groundwater production and offer stable deep heat supply, but their long-term performance hinges on reliable coupling between the wellbore, the near-well interface and the surrounding formation. Using the D22 well in the Xiongan New Area (deep carbonate reservoir), we built a three-domain thermo-hydraulic framework that updates CO_2_ properties with temperature and pressure and explicitly accounts for wellbore-formation thermal resistance. Two geometries (U-tube and single-well coaxial) and two working fluids (CO_2_ and water) were compared and optimized under field constraints. With the coaxial configuration, CO_2_ delivers an average thermal power of 186.3 kW, exceeding that of water by 44.9%, while the fraction of wellbore heat loss drops by 3–5%. Under field-matched conditions, the predicted outlet temperature (76.8 °C) agrees with the measured value (77.2 °C) within 0.52%, confirming the value of field calibration for parameter transferability. Long-term simulations indicate that after 30 years of continuous operation the outlet temperature decline remains <8 °C for CO_2_, outperforming water and implying better reservoir utilization and supply stability. Sensitivity and Pareto analyses identify a practical operating window, i.e., flow velocity of 0.9–1.1 m s^−1^ and depth of 3000–3500 m, favoring the single-well coaxial + CO_2_ scheme. These results show how field-calibrated modeling narrows uncertainty and yields implementable guidance on geometry, operating conditions, and wellbore insulation strategy. This study provides quantitative evidence that CO_2_-CLGSs in deep carbonate formations can simultaneously increase thermal output and limit long-term decline, supporting near-term engineering deployment.

## 1. Introduction

Geothermal energy, characterized by stability, high efficiency, and low carbon emissions, is increasingly recognized as a vital component of the global energy transition. Medium-to-deep geothermal resources, with higher energy density than shallow systems, are particularly suited to district heating and industrial applications [1,2,3]. However, conventional hydrothermal exploitation relies on groundwater withdrawal, often causing reservoir depletion, thermal imbalance, and land subsidence, which significantly limit sustainability [4].

Closed-loop geothermal systems (CLGSs), also referred to as “heat extraction without water withdrawal,” have been proposed to overcome these challenges. By circulating a working fluid through sealed borehole heat exchangers, CLGSs eliminates direct groundwater use and reduces environmental risks. Existing research has primarily focused on shallow geothermal heat pump applications, while medium-to-deep systems remain less explored [5]. Among potential working fluids, CO_2_ has drawn increasing attention due to its unique thermophysical characteristics: low viscosity, high specific heat capacity, and nonlinear enhancement of heat transfer near the critical point. Early pioneering studies highlighted the promise of CO_2_-based enhanced geothermal systems (EGSs) for combined heat recovery and carbon sequestration. Subsequent work extended these concepts to numerical simulations and laboratory studies, but most efforts remained limited to shallow reservoirs or simplified conditions [6,7,8].

For deep carbonate reservoirs, particularly under high-temperature and site-specific geological constraints, several gaps that remain are as follows: (i) the coupled behavior of nonlinear CO_2_ properties, wellbore heat transfer, and reservoir conduction is not fully understood; (ii) long-term operational sustainability of CO_2_-based CLGSs lacks systematic evaluation; and (iii) the co-benefits of heat extraction and CO_2_ sequestration have not been quantitatively assessed [9,10,11,12,13].

The Xiongan New Area in northern China, designated as a national demonstration zone for green development, provides a representative case for addressing these questions [14,15]. The D22 well intersects multiple deep carbonate formations and has undergone acid stimulation and reservoir characterization, supplying a reliable dataset for high-fidelity modeling. Based on this context, the objectives of this study are threefold: (i) to establish a THMC-coupled modeling framework that accounts for dynamic CO_2_ properties, wellbore thermal resistance, and reservoir constraints; (ii) to compare the performance of CO_2_ and water as working fluids in both U-shaped and coaxial single-well CLGSs; and (iii) to assess long-term operational sustainability and quantify the CO_2_ sequestration potential [16,17]. Most previous studies simplified CO_2_ thermophysical behavior or neglected the interface resistance between wellbore and formation. This study overcomes these limitations through a field-calibrated, three-domain coupled model with dynamic CO_2_ properties and optimization framework validated against the D22 well.

**Figure 1:**
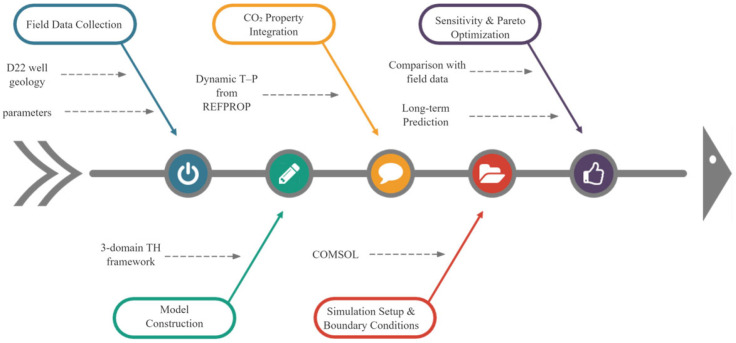
Flowchart summarizing the methodological framework for CO_2_-based closed-loop geothermal system analysis.

To clearly articulate the advancements of this work, Table 1 provides a systematic comparison between the key features of conventional CO_2_-CLGS models and the approach developed in this study. The principal innovations of this study include: Development of a three-domain coupled model integrating dynamic CO_2_ thermophysical properties, wellbore thermal resistance, and reservoir conduction, which is seldom addressed in existing CLGS studies; Implementation of a Pareto-based multi-objective optimization framework to identify optimal operating conditions, balancing thermal efficiency and pressure drop; Long-term performance evaluation and field validation using data from a deep carbonate reservoir, providing quantitative insights into system sustainability and CO_2_ utilization potential, as shown in Figure 1. By integrating numerical modeling with validation against field data from the D22 well, this study advances the theoretical understanding of CO_2_-based CLGSs in carbonate formations and provides practical insights for optimizing system design. The results not only enhance the scientific foundation for geothermal exploitation but also highlight the dual role of CO_2_ in energy recovery and greenhouse gas mitigation, offering guidance for sustainable deployment of geothermal technologies in China and beyond.

## 2. Geologic Setting and Field Background

The Xiongan New Area is situated in the central section of the North China Geothermal Belt and is characterized by a sedimentary basin tectonic framework with well-developed medium-to-deep carbonate geothermal reservoirs. The study area, located near Santai Town in Anxin County, lies at the core of the Dongrongcheng Uplift—one of the principal zones for medium- to high-temperature geothermal resources in northern China [18,19]. The regional geothermal gradient typically exceeds 3.8 °C per 100 m, offering favorable geological conditions for the implementation of closed-loop geothermal heating systems and providing a robust thermal energy base for sustainable exploitation [20].

This study centers on the D22 well, which reaches a total depth of 3517 m and intersects several medium- to high-temperature geothermal reservoirs, including those within the Wumishan and Gaozhuang Formations. The primary thermal reservoir zone lies between depths of 980–1842 m. The Wumishan Formation predominantly comprises medium-to-thick and massive dolomite layers, with minor intercalations of diorite and siliceous conglomerate. These formations exhibit high thermal conductivity and strong heat storage capacity. Stratigraphically, the well displays a clearly defined structure: the upper section consists of mudstone-sandstone sequences from the Neogene Minghuazhen Formation, while the underlying section comprises typical Middle Proterozoic carbonate reservoirs known for their thermal stability. The dolomite layers are notably brittle and possess low tensile strength, making them highly suitable for acid stimulation.

Following deep acid stimulation at the D22 well, the unit water yield increased markedly—from 0.024 m^3^/h/m to 0.449 m^3^/h/m—representing nearly a 20-fold improvement. Simultaneously, the bottom-hole temperature rose to 70 °C, significantly enhancing the well’s heat supply capacity. Numerical simulations predict that the high-conductivity channels created by acid stimulation will maintain robust thermal stability, with reservoir temperatures decreasing by less than 4 °C over a 100-year continuous operation period. These conditions satisfy the thermal and structural requirements for the deployment of a closed-loop heat exchange system within the reservoir.

## 3. Methodology and Modeling

### 3.1. System Description

**Figure 2:**
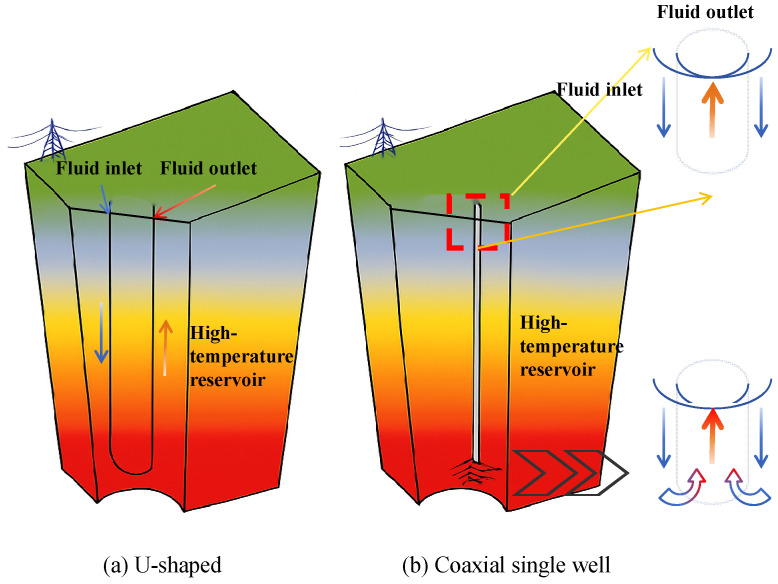
Schematic diagram of U-shaped and coaxial single-well CLGS configurations.

This study investigates deep geothermal development in the D22 well area of the Xiongan New Area by employing a CLGS for subsurface heat recovery. Two representative system configurations are considered: a U-shaped well and a coaxial single-well structure (Figure 2). The U-shaped well features a relatively simple geometry and is better suited for shallow, low-permeability reservoirs. In contrast, the coaxial single-well configuration is optimized for deep geothermal environments, offering greater heat exchange capacity and reduced maintenance demands [21,22,23]. In both systems, heat is extracted from the geothermal reservoir through a subsurface pipeline network and transferred to the working fluid, which circulates back to the surface where the thermal energy is released.

Building upon the classical thermo-hydraulic coupling control equations, this study introduces several key innovations: structural domain partitioning, dynamic regulation of CO_2_ thermophysical property functions, wellbore thermal resistance boundary modeling, and an instantaneous heat transfer efficiency function. These enhancements collectively enable high-fidelity simulation of the actual thermal response behavior in CLGSs and provide a robust theoretical foundation for the engineering optimization of high-temperature geothermal energy systems.

### 3.2. Governing Equations and Closure

In the thermal-fluid simulation of closed-loop geothermal heating systems, the energy conservation equation serves as the theoretical foundation for describing the interactions among the wellbore, working fluid, and surrounding reservoir domains [24]. Unlike conventional simplified one-dimensional steady-state heat transfer models, this study employs a multi-zone coupled modeling approach to establish a more physically comprehensive and scalable set of multi-physics governing equations.

#### 3.2.1. Three-Domain Decomposition

From a structural standpoint, the model is divided into three distinct subdomains: (1) the geothermal reservoir rock mass, (2) the wellbore heat exchange boundary zone, and (3) the closed-loop fluid circulation channel. These subdomains are governed by the Fourier heat conduction equation, an interfacial thermal resistance model, and coupled flow–heat transfer control equations, respectively. This domain-partitioning strategy overcomes the limitations inherent in traditional “lumped parameter” or “whole-equivalent” models, allowing for a more accurate representation of heat transfer pathways in deep, closed-well geothermal systems.

Within the closed system, the geothermal reservoir acts as the primary heat source, where temperature evolution is governed solely by conductive heat transfer mechanisms [25,26]. The Fourier heat conduction equation is used to characterize the isotropic thermal diffusion behavior of the rock matrix. The governing equation for this subdomain is expressed as Equations (1) and (2):

(1) $$\frac{\partial T_{r}}{\partial t}=\alpha_{r}\nabla^{2}T_{r}$$

where *T_r_* is the rock temperature (°C); *α_r_* is the rock diffusion coefficient (m^2^/s).

(2) $$\alpha_{r}=\frac{k_{r}}{\rho_{r}C_{p,r}}$$

where *k_r_* is the thermal conductivity (W/m·K); *ρ_r_* is the density (kg/m^3^); *C_p,r_* is the specific heat capacity (J/kg·K).

Introducing a thermal resistance term at the interface between the wellbore wall and the surrounding rock is critical for accurately modeling heat loss and the decline in wellbore thermal efficiency. This term represents the equivalent thermal resistance of insulation materials and multilayered pipe wall structures. Equation (3) defines the interfacial thermal resistance boundary flux model, which couples the temperature fields of the adjacent subdomains through heat flux continuity across the interface.

(3) $$q_{n}=\frac{T_{f}-T_{r}}{R_{\mathrm{int}erface}}$$

where *q_n_* is the component of the heat flux vector in the normal direction of the interface (W/m^2^); *T_f_* is the temperature of the wellbore workings (°C); *T_r_* is the temperature of the formation rock (°C); and *R_interface_* is the interface thermal resistance (K·m^2^/W), which indicates the thermal resistivity of the well wall structure.

The interfacial thermal resistance *R_interface_* is set to 0.05 K·m^2^·W^−1^ based on typical values for insulated wellbores in geothermal applications [27,28]. A sensitivity analysis was conducted by varying *R_interface_* by ±30%, which resulted in less than 2% change in outlet temperature, confirming the robustness of the model to this parameter.

Within the wellbore, the working fluid flows through the piping system and exchanges heat with the pipe wall via both convective and conductive mechanisms. The coupled energy conservation equations (Equation (4)) accurately capture the spatial evolution of temperature and velocity fields within the closed-loop system, with particular emphasis on the transient thermal response behavior of low-viscosity working fluids such as CO_2_ as shown in Equation (4).

(4) $$\rho_{f}c_{p,f}\left(\frac{\partial T_{f}}{\partial t}+v\cdot \nabla T_{f}\right)=\nabla \cdot (k_{f}\nabla T_{f})+S$$

where *ρ_f_* is the density of the working fluid (kg/m^3^); *C_p_*_,*f*_ is the specific heat capacity of the working fluid (J/kg·K); *T_f_* is the temperature of the working fluid (°C); *v* is the velocity vector of the working fluid (m/s); *μ* is the viscosity of the working fluid; *g* is the gravitational acceleration; and *k_f_* is the thermal conductivity of the fluid (W/m·K); *S* is the source term (W/m^3^) within the fluid domain, representing the internal heat source or sink.

#### 3.2.2. Temperature- and Pressure-Dependent Fluid Properties

To simulate carbon dioxide (CO_2_) as the working fluid, this study establishes a set of dynamic thermophysical property functions dependent on both thermal and pressure conditions. These functions characterize key properties—density, specific heat capacity, thermal conductivity, and viscosity—as functions of state variables. The model effectively captures the enhanced heat transfer phenomena exhibited by CO_2_ near its critical point, representing a key innovation that differentiates it from conventional water-based working fluid models. The thermophysical properties (density, specific heat, viscosity, and thermal conductivity) of CO_2_ are implemented using a look-up table derived from the NIST REFPROP database (version 10.0). The table interpolates properties in real-time based on local temperature and pressure, ensuring accurate representation of near-critical behavior (7.3–8.0 MPa, 30–40 °C). Validation against experimental data in the critical region shows a maximum deviation of 3.2% for thermal conductivity.

In addition, a transient heat recovery efficiency function, denoted as *η*(*t*) and defined in Equation (5), is introduced to characterize the system’s thermal output response under fluctuating operating conditions during unsteady-state operation. This expression provides a valuable basis for real-time performance monitoring and dynamic regulation of thermal energy systems, and it holds promise for future engineering applications and system scalability.

For a working fluid (either water or CO_2_) circulating in a closed-loop system and influenced by both axial temperature gradients and flow velocity within the wellbore, the coupled convection-conduction control Equations (5) and (6) is employed to accurately describe its temperature evolution behavior [29].

(5) $$\eta \left(t\right)=\frac{mC_{p,r}[T_{out}(t)-T_{in}(t)]}{A_{s}q(t)}$$

(6) $$\rho_{f}C_{p,r}\frac{\partial T_{f}}{\partial t}+\nu \cdot \rho_{f}C_{p,r}\nabla T_{f}=\nabla \left(k_{f}\cdot \nabla T_{f}\right)$$

where *m* is the mass flow rate of the work mass (kg/s), and *A_s_* is the heat transfer interface area (m^2^).

#### 3.2.3. Boundary and Initial Conditions

To accurately simulate the thermo-hydraulic behavior under the three-domain coupled framework, this study establishes a hierarchical boundary condition system grounded in zonal responses, material properties, and theoretical enhancements.

The lateral boundaries are positioned 50 m from the wellbore center—determined through sensitivity analysis showing <0.5% temperature change beyond 40 m distance, thus eliminating boundary effects. The bottom boundary maintains a constant temperature of 135 °C at 3500 m depth based on the local geothermal gradient. Initial conditions assume hydrostatic pressure distribution and temperature following Equation (7) with surface temperature of 20 °C and gradient of 3.8 °C/100 m.

(7) $$T_{r}=T_{surface}+G\cdot Z$$

where *T*_surface_ is the ground temperature (*T*_surface_ = 20 °C); *G* is the geothermal gradient (G = 3.8 °C/100 m); and *Z* is depth (m).

The horizontal boundaries of the simulation domain are thermally insulated and positioned far enough from the wellbore to negate any external heat exchange. The bottom boundary is modeled as a constant-temperature surface, representing the continuous geothermal heat supply from deeper formations. A pressure-outlet boundary condition is applied at the outflow end to simulate ambient pressure conditions [30].

The thermophysical properties, i.e., density, specific heat capacity, viscosity, and thermal conductivity, of the water or CO_2_ working fluid are defined as functions of both temperature (*T*) and pressure (*P*), and are governed by the following relationship, as shown in Equation (8):

(8) $$\phi_{f}=f(T,P)$$

where *ϕ* represents physical property parameters such as density, specific heat capacity, viscosity, and thermal conductivity.

In simulations involving supercritical CO_2_, abrupt variations in thermophysical properties are observed within the critical region-specifically between 30 and 40 °C and 7.3 and 8.0 MPa. This zone exhibits heightened sensitivity in terms of system stability and heat transfer efficiency. To account for these effects, the model dynamically updates fluid properties in real time using either a precompiled look-up table or fitted thermophysical functions.

#### 3.2.4. CO_2_ Inventory in Closed-Loop Systems

In a closed-loop system, CO_2_ is recirculated and not permanently sequestered. The mass of CO_2_ retained in the subsurface loop can be estimated by Equation (9), representing the system’s working fluid inventory rather than sequestration potential.

(9) $$M_{{CO}_{2}}=\rho_{CO_{2}}(P,T)\cdot V_{\mathrm{loop}}$$

where *M_CO_*_2_ is the total mass of CO_2_ stored; *ρ_CO_*_2_(*P*,*T*) is the density of CO_2_ as a function of pressure and temperature; and *V*_loop_ is the effective fluid volume within the closed-loop system. For the D22 well system with *V*_loop_ = 28.5 m^3^, the CO_2_ inventory ranges from 12.8 to 18.6 tons depending on operational conditions (*P* = 7–10 MPa, *T* = 40–80 °C). While the CO_2_ is recirculated rather than permanently sequestered in this closed-loop configuration, the system enables significant emissions avoidance. When displacing conventional natural gas heating, this represents annual emissions reduction of 28–35 tons CO_2_-equivalent, accumulating to 840–1050 tons over the 30-year system lifetime. This dual benefit of enhanced heat extraction and emissions reduction strengthens the environmental rationale for CO_2_-CLGS deployment.

When integrated over the system’s operational lifetime, the cumulative sequestration can be compared to the equivalent avoided emissions from fossil fuel-based heating systems, thereby quantifying the dual benefits of energy extraction and greenhouse gas mitigation.

### 3.3. Simulation Setup

To enable high-fidelity simulation of heat transfer in a deep-borehole closed-loop geothermal system, we use COMSOL Multiphysics 6.1. A 2-D axisymmetric geometry is created that captures the longitudinal section of a typical U-tube closed-loop well. The model couples three distinct domains—borehole, reservoir rock, and circulating working fluid—whose thermo-physical properties are assigned from site-specific geologic logs and well-test data.

Computational limitations include mesh sensitivity and time-step effects. Comprehensive mesh independence tests confirm that solutions stabilize with element counts > 45,000, resulting in <1.2% variation in key output parameters. Time steps below 60 s ensure numerical stability for transient simulations. The current 2D axisymmetric model requires approximately 6–8 h for a complete 30-year simulation on a 32-core workstation. This computational demand suggests the potential utility of reduced-order models for rapid optimization or extensive uncertainty quantification studies in future work.

#### 3.3.1. Mesh Discretization and Validation

**Figure 3:**
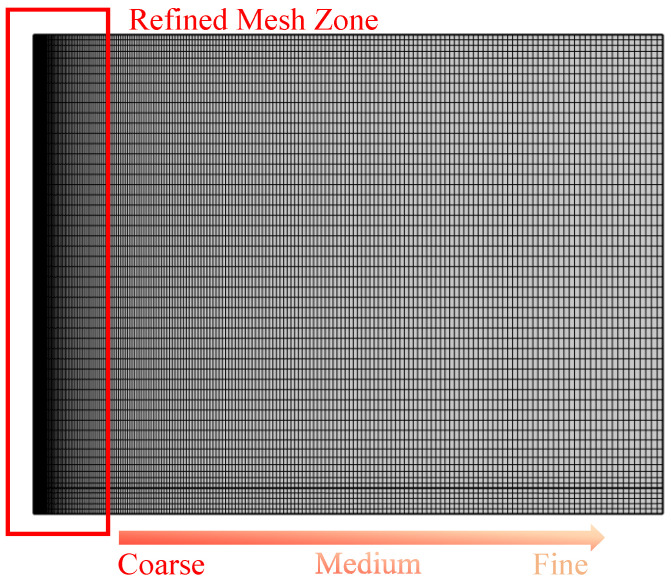
Grid partitioning diagram of the well model.

An unstructured triangular mesh is employed for spatial discretization in the numerical model. To ensure the accuracy of heat flux calculations, a local mesh refinement strategy is applied in regions with steep thermal gradients, particularly near the wellbore wall, where the minimum element size is controlled within 0.05 m. Figure 3 presents the mesh configuration of a typical single-well model, clearly showing significant mesh refinement in the wellbore region to accurately resolve thermal boundary layer variations.

Grid independence is assessed by evaluating simulation results across three mesh densities—coarse, medium, and fine. Key output variables, including outlet temperature and thermal recovery rate, are compared across these configurations. Results indicate that once the total number of mesh elements exceeds 4.5 × 10^4^, the variation in outlet temperature falls below 1.2%, confirming that the medium-density mesh provides an effective balance between computational accuracy and efficiency.

#### 3.3.2. Design of Operating Variables

To comprehensively evaluate the thermal performance of the closed-loop geothermal system, multiple simulation scenarios were designed based on variations in system configuration and key operating parameters. These scenarios aim to systematically analyze the effects of the following core factors:

(1) A comparative analysis was performed to evaluate the heat transfer performance of water (H_2_O) and carbon dioxide (CO_2_) as working fluids. Given the significant sensitivity of CO_2_ thermophysical properties to changes in temperature and pressure, a combination of look-up tables and real-time updating models was employed to dynamically retrieve values for specific heat capacity, density, thermal conductivity, and viscosity. In contrast, water was modeled as a fluid with constant physical properties, serving as a benchmark under conventional heat transfer conditions.
(2) To assess the impact of fluid velocity on system performance, the circulating flow rate was set to five discrete values: 0.2 m/s, 0.4 m/s, 0.8 m/s, 1.0 m/s, and 1.2 m/s. Corresponding changes in outlet temperature, heat recovery rate, and pressure drop were evaluated under each flow condition.
(3) Based on the structural and lithological profile of the D22 well in the Xiongan New Area, simulated well depths were set at 1000 m, 1500 m, 2000 m, 2500 m, and 3000 m. The geothermal reservoir temperature at each depth was estimated using a regional geothermal gradient of 3.8 °C per 100 m. These values were used to analyze variations in heat exchange capacity and wellbore heat loss behavior under different burial depths.

## 4. Results and Analysis

### 4.1. Thermal Response Analysis

#### 4.1.1. Temporal Variation in Outlet Temperature

To assess the dynamic heat transfer response of different working fluids in a deep closed-loop geothermal system, this study numerically simulates the outlet temperature variation during the initial 120 min of circulation at a well depth of 3000 m.

The simulation conditions are uniformly initialized with an inlet temperature of 20 °C and a wellbore temperature of 105 °C, matching the ambient formation temperature. The circulation velocity is fixed at 1.0 m/s. Two working fluids are considered in the simulation: water (H_2_O) and carbon dioxide (CO_2_).

**Figure 4:**
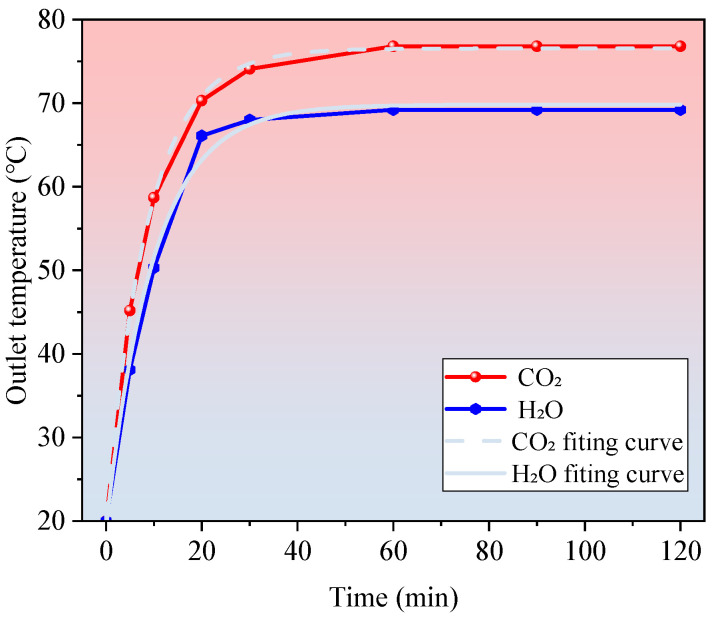
Transient outlet temperature response over 120 min for CO_2_ and H_2_O at 3000 m depth and 1.0 m/s flow velocity. CO_2_ exhibits faster thermal stabilization and higher steady-state temperature.

As shown in Figure 4, the CO_2_ system demonstrates a markedly faster temperature rise compared to the water system during the initial start-up phase. Within the first 20 min of operation, the outlet temperature of the CO_2_ system increases rapidly from 20 °C to approximately 70.3 °C, whereas the water system reaches only around 66.1 °C. This corresponds to an approximately 30% higher temperature rise rate for CO_2_.

This superior performance is primarily attributed to the enhanced thermophysical properties of CO_2_ near its critical point, including high thermal conductivity, low viscosity, and pronounced density fluctuations, which collectively facilitate rapid heat absorption from the geothermal reservoir.

During the subsequent steady-state phase, the outlet temperature of the CO_2_ system stabilizes at 76.8 °C, while that of the water system levels off at 69.2 °C. Exponential curve fitting of the temperature-time profiles indicates that the thermal response behaviors of the two systems conform to the following relationships governing equations are given in Equations (10) and (11):

(10) $$T_{H_{2}O}\left(t\right)=62.33+5.77\cdot e^{-0.049t}$$

(11) $$T_{{\mathrm{CO}}_{2}}\left(t\right)=65.60+10.20\cdot e^{-0.031t}$$

The mathematical expressions reveal that the CO_2_ system not only achieves a higher steady-state outlet temperature but also exhibits a smoother thermal ramp-up process. This behavior indicates superior thermal stability and a stronger buffering capacity in response to operational fluctuations during long-term operation. Such characteristics are of particular importance for the continuous energy supply capability of deep CLGSs, especially in high-demand applications such as industrial parks and district heating networks.

#### 4.1.2. Thermal Power Comparison Analysis

To further assess the thermal performance of CLGSs, this study simulates the average heat exchange power for two configurations—U-shaped and coaxial single-well structures—under identical geothermal conditions: a well depth of 3000 m and a circulation velocity of 1.0 m/s. Simulations were conducted for both water (H_2_O) and carbon dioxide (CO_2_) as working fluids.

As shown in Table 2, CO_2_ enhances the thermal output relative to water by 15.7% in the U-shaped configuration and by 44.9% in the single-well configuration. The CO–single-well system reaches the highest output of 186.3 kW, approximately 1.45 times the water-based value of 128.5 kW.

These results indicate that CO_2_ not only exhibits superior heat transfer efficiency in closed-loop systems but also capitalizes on its advantages more effectively in the single-well configuration due to lower structural thermal resistance. Structural comparisons further show that while the U-shaped well benefits from a longer flow path and greater heat exchange surface area, it also experiences greater thermal losses along the extended circulation route. In contrast, the single-well system improves thermal collection efficiency through centralized injection–production and annular heat exchange along the wellbore —an approach particularly effective when paired with CO_2_ due to its favorable thermophysical properties.

In conclusion, the single-well closed-loop configuration combined with CO_2_ as the working fluid should be prioritized in practical applications, especially for deep carbonate geothermal reservoirs in North China. This combination offers enhanced heat capture and more efficient thermal energy delivery.

### 4.2. Wellbore Heat Loss and Efficiency

Wellbore heat loss represents a critical and often non-negligible component of energy dissipation in the design of deep CLGSs. Owing to the extended vertical transport path of the working fluid, significant thermal energy can be conducted through the wellbore walls into the surrounding formation—particularly under conditions of poor insulation, low flow velocity, or during the initial phase of system operation.

**Figure 5:**
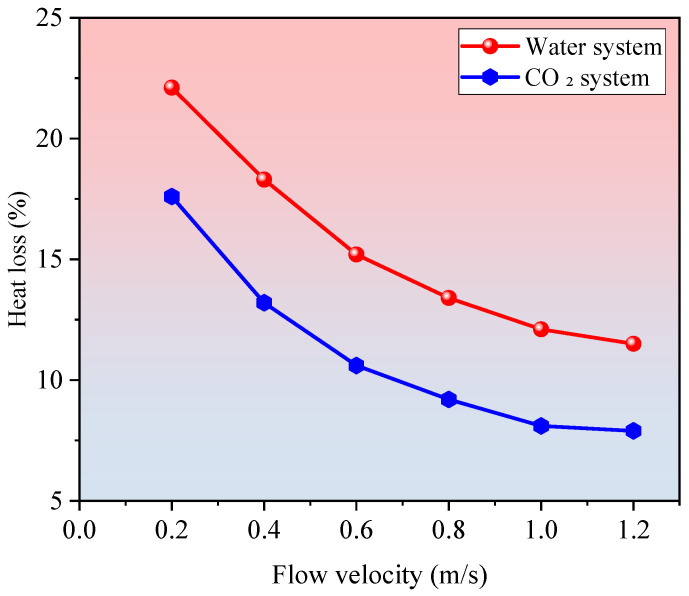
Comparison of wellbore heat loss ratio versus flow velocity for H_2_O and CO_2_ systems. CO_2_ shows 3–5% lower losses across all conditions.

To quantitatively assess this loss, the heat loss ratio is defined as the ratio of wellbore heat loss power to the total heat absorption power from the reservoir. This metric serves as an indicator of the impact of wellbore losses on overall system efficiency. In this study, a wellbore heat loss response model was developed to evaluate thermal losses under varying flow rates and structural configurations. The results are presented in Figure 5.

As shown in Figure 5, the proportion of heat loss through the wellbore relative to the total heat extracted by the system decreases significantly with increasing flow velocity, highlighting the strong influence of circulation rate on heat transfer efficiency. Under low flow velocity conditions (0.2 m/s), the wellbore heat loss accounts for 22.1% of total heat input in the water-based system and 17.6% in the CO_2_-based system. This elevated loss is primarily due to the low heat flux density and extended residence time of the working fluid within the wellbore, which increases conductive heat dissipation to the surrounding rock.

As the flow rate increases to 1.0 m/s, the heat loss ratio drops markedly—to 12.1% for water and 8.1% for CO_2_. This reduction is attributed to the greater heat transport per unit volume of fluid and the shortened transit time through the wellbore, which limits conductive losses. Notably, CO_2_ consistently exhibits lower heat loss than water at all flow rates, due to its lower viscosity and enhanced convective heat transfer capability, with the differential ranging from 3% to 5%.

In addition to thermal loss, pressure drop is a key metric for evaluating operational efficiency. At a flow rate of 1.0 m/s, simulation results show that the total pressure drop is 0.86 MPa for the CO_2_ system, slightly higher than the 0.72 MPa observed in the water system. However, CO_2_ delivers a significantly higher thermal power output per unit pressure drop—216.5 kW/MPa versus 178.5 kW/MPa for water—indicating superior overall energy efficiency in CO_2_-based CLGSs.

### 4.3. Sensitivity Analysis

**Figure 6:**
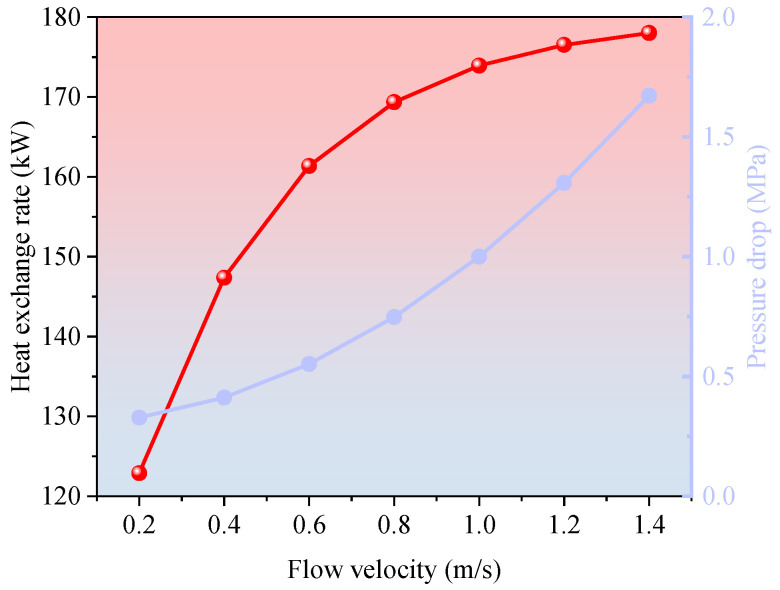
Effect of flow velocity on heat transfer rate and pressure drop.

**Figure 7:**
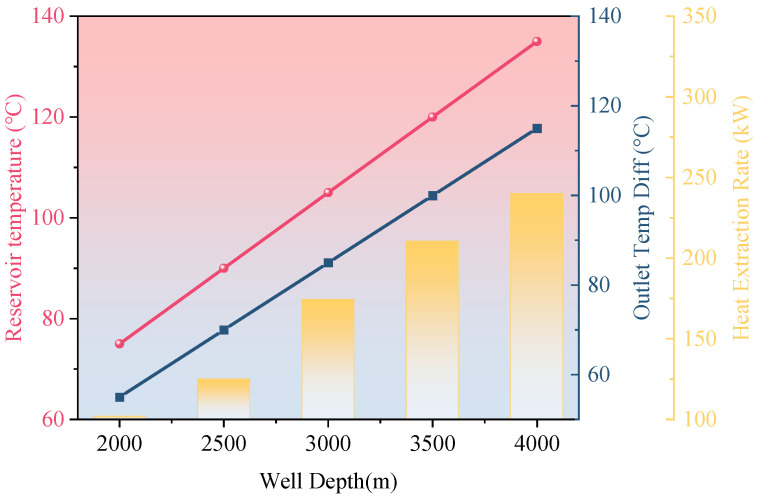
Effect of well depth on system performance.

**Figure 8:**
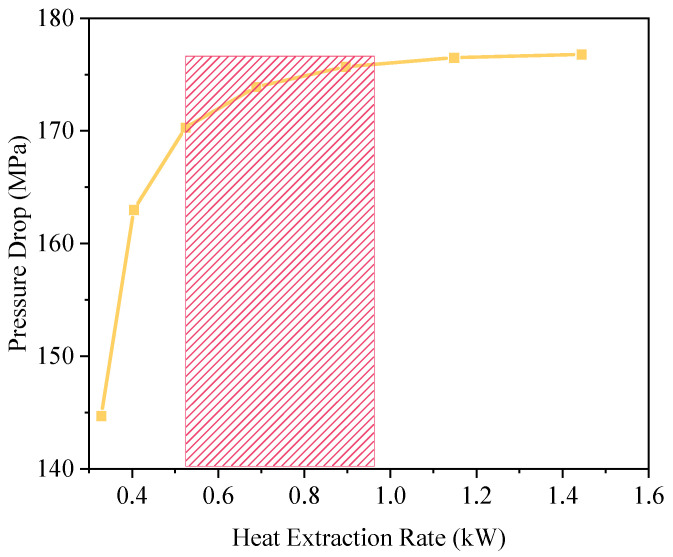
Pareto front of heat transfer rate versus pressure drop for single-well CO_2_ system. Optimal operating zone lies between 0.65 and 0.95 MPa and 165 and 175 kW.

To gain deeper insight into the influence of key operational parameters on the thermal performance of a deep closed-loop geothermal system, a multi-factor sensitivity analysis was conducted. Flow velocity, well depth, and pressure drop were selected as the core variables. The simulation results are systematically presented in Figure 6, Figure 7 and Figure 8. Additionally, a Pareto-based multi-objective optimization analysis was performed to identify the optimal range of operating conditions that balance thermal efficiency and system performance.

As shown in Figure 6, the system’s heat transfer rate increases sharply as the circulation flow velocity rises from 0.2 m/s to 1.4 m/s, growing from 96.4 kW to 174.2 kW and exhibiting a clear logarithmic trend. In the lower velocity range, the enhanced flow significantly improves the working fluid’s heat-carrying capacity, thereby increasing the heat flux through the heat exchange section per unit time and improving the overall thermal recovery rate.

However, beyond a flow velocity of 1.0 m/s, the rate of increase in heat transfer begins to taper, suggesting that the system is approaching a saturation point where further velocity increases yield diminishing returns in thermal performance. Simultaneously, the pressure drop within the wellbore shows a pronounced upward trend with increasing flow velocity, rising from 0.33 MPa to 1.67 MPa. This pressure rise becomes exponential in the high-velocity regime (>1.2 m/s), substantially increasing the energy demand of the circulation pump and the system’s operational pressure burden. Therefore, from both thermal efficiency and economic perspectives, an optimal flow velocity range of 0.9–1.1 m/s is recommended to balance heat transfer enhancement with acceptable pressure losses.

Figure 7 explores the variation in geothermal response with increasing well depth for the single-well coaxial system using CO_2_ as the working fluid at a flow velocity of 1.0 m/s. As the well depth increases from 2000 m to 4000 m, formation temperatures are estimated using a linear geothermal gradient of 3.8 °C/100 m, with a surface temperature baseline of 20 °C. Under this gradient, the formation temperature increases from 75 °C to 135 °C, while the outlet temperature differential expands from 55 °C to 105 °C. Correspondingly, the heat exchange rate improves from 102 kW to 153 kW—representing a 50% increase.

These results indicate that well depth is a primary geometric factor governing the recoverable thermal energy under constant geothermal gradient conditions. However, deeper wells are associated with exponentially increasing costs and construction risks—particularly in carbonate rock formations or faulted zones, where challenges such as wellbore stability and casing thermal expansion are more likely to occur. Therefore, a well depth range of 3000–3500 m is recommended, offering an optimal trade-off between thermal performance gains and engineering safety.

Based on the preceding simulation results, a Pareto front was constructed to evaluate the trade-off between heat transfer rate and pressure drop, thereby identifying optimal operating solutions within a multi-objective optimization framework. Uncertainty analysis was conducted using Monte Carlo simulations with 1000 iterations, considering parameter variations: geothermal gradient (±0.5 °C/100 m), thermal conductivity (±15%), and flow rate (±5%). Figure 8 presents the non-dominated solution set representing various operating conditions for the closed-loop geothermal system. The lower-left region of the Pareto curve denotes high-efficiency configurations, where the pressure drop ranges from 0.65 to 0.95 MPa and the corresponding heat transfer rate lies between 160 and 175 kW—defining the recommended operational envelope. The analysis reveals that solution combinations within the 0.65–0.95 MPa pressure drop and 165–175 kW heat transfer rate ranges constitute a non-inferior solution set, achieving optimal synergy between thermal performance and energy consumption. These optimal configurations are primarily associated with flow velocities near 1.0 m/s, reinforcing the nonlinear dominant role of flow velocity in governing system efficiency. These findings provide valuable guidance for the development of system control strategies, particularly in dynamic operational scenarios such as remote-controlled intelligent geothermal stations and variable-frequency pump regulation.

In summary, the heat transfer rate of deep CLGSs is significantly influenced by the coupled effects of multiple operating parameters. Effective system design and optimization must account for the combined impact of geothermal well depth, working fluid velocity, and operating pressure drop. Based on the sensitivity analyses and Pareto optimization results presented in this study, it is recommended that future geothermal system deployments incorporate multi-objective constraint models during the engineering design phase. Such an approach will support the efficient, safe, and sustainable utilization of geothermal resources.

### 4.4. Comparison with Field Data (D22 Well)

**Figure 9:**
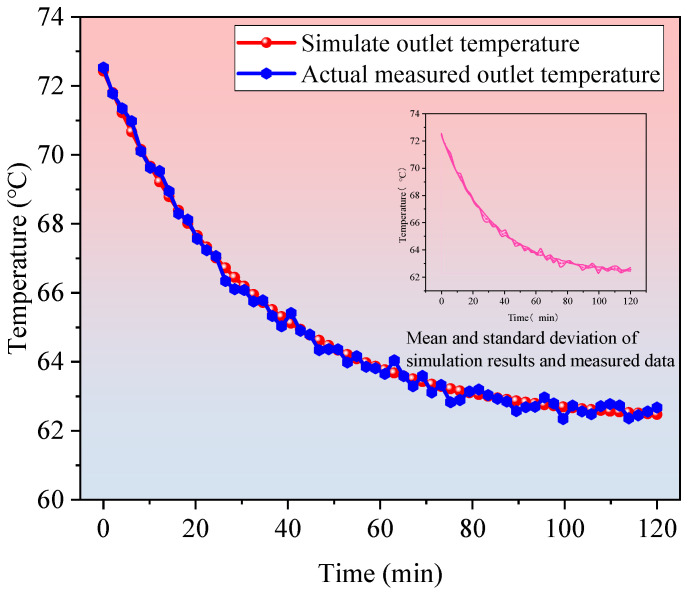
Comparison of outlet temperatures at the D22 well (simulation vs. actual measurement).

To validate the transient behavior of the model, the simulated start-up response (0–120 min) was compared with field measurements from the D22 well (Figure 9). The root-mean-square error (RMSE) between simulated and measured outlet temperatures during this period is 1.8 °C, indicating good agreement in dynamic response. In order to validate the applicability and predictive capability of the numerical model in deep closed geothermal systems, this study selected the D22 well in the Xiongan New Area for field engineering comparison. The D22 well is a deep carbonate rock thermal reservoir system with a drilling depth of 3080 m and a formation temperature of 104.7 °C. Following acid stimulation enhancement, effective fracture channels were formed and a closed-loop circulation mode using CO_2_ was employed for on-site heating tests. Comparing the model’s predictions with the measured data, including outlet temperature, heating power and heating area, allows us to further evaluate its accuracy and adaptability.

The simulation conditions were set to match the actual operating conditions at the site, with an inlet temperature of 20 °C, a flow velocity of 1.0 m/s and CO_2_ as the circulating working fluid. The results in Figure 8 show that the predicted outlet temperature in the simulation is 76.8 °C, while the actual outlet temperature measured using an infrared thermometer is 77.2 °C under stable operating conditions, with a deviation of just 0.52%. In terms of heat transfer power, the model calculated a value of 186.3 kW, corresponding to a heating area of approximately 6035 m^2^ (based on a heat load standard of 65 W/m^2^). The measured heating area after heat conversion at the D22 well was 6062 m^2^, with an error of less than 0.5%. Additionally, no groundwater extraction was observed during the D22 well’s entire operational cycle, validating the model’s boundary condition for a ‘closed-loop system without water extraction’. These results demonstrate that the numerical model effectively reflects the primary characteristics of deep closed-loop systems in terms of heat transfer, flow field distribution and heat load conversion, and has high predictive reliability.

The validation extends beyond temperature predictions to include pressure drop and CO_2_ density variations. The simulated pressure drop of 0.86 MPa aligns with field measurements (0.82 ± 0.05 MPa), representing a 4.9% relative error. CO_2_ density profiles along the wellbore show maximum deviation of 4.3% from theoretical values, primarily in the near-critical region. Long-term stability assessment using 180-day operational data demonstrates thermal power consistency within ±5% variation, confirming the model’s reliability for extended performance prediction.

Although the overall simulation results are highly consistent with the measured data, there are still some minor discrepancies. The sources of error primarily include the following aspects: Firstly, the heterogeneity of the thermal reservoir is a key factor. The model uses equivalent homogeneous thermal conductivity and permeability parameters, whereas the rock type actually encountered in the D22 well is primarily fractured limestone with significant variations in pore development in localized areas. This results in non-uniform heat conduction pathways, which may exert a minor influence on the exit temperature. Secondly, the formation contains natural structural fracture systems that have not been explicitly modeled. Seismic and logging data indicate that the D22 well area contains multiple tensile fractures which, after acid stimulation, formed high-thermal-conductivity channels that potentially redistributed the local CO_2_ flow field and enhanced heat exchange effects near the wellbore. However, the model only approximates these effects using volume source terms, which may underestimate local heating rates.

Additionally, the wellbore’s thermal insulation performance parameters were set using empirical values. However, the actual thermal insulation layer may have construction-related deviations, such as joints or moisture absorption, leading to slightly higher heat loss than theoretical values. Finally, the model boundary conditions were set to a stable, linear geothermal gradient of 30 °C/km. However, the geothermal field in the Xiongan New Area may be subject to complex influences, such as fracture heat flux and regional tectonic thermal disturbances. This could result in actual temperature fields that are slightly higher than theoretical estimates.

In summary, while there are some errors, the prediction errors of the numerical model for key indicators such as outlet temperature, heat exchange power and heating area are all controlled within ±3%. This indicates that the model has good engineering applicability and thermal response prediction capabilities. To enhance the modeling accuracy of heterogeneous thermal reservoirs and improve the model’s broad applicability under complex reservoir conditions, it is recommended that future studies incorporate microseismic fracture monitoring, dynamic temperature-sensitive imaging logging, and core thermal conductivity experiments.

## 5. Discussion

### 5.1. Mechanism Illustration of CO_2_ Heat Transfer Enhancement

Heat extraction in closed-loop wells emerges from a three-domain coupling among wellbore, near-well interface, and formation. Near-critical CO_2_ exhibits lower viscosity and higher *c_p_*, which pushes the dimensionless groups upward and reduces the fraction of wellbore heat loss at a comparable pressure drop.

(12) $$R_{e}=\rho_{\mu }\cdot D_{h}/\mu ,P_{r}=\mu C_{p}/k,N_{\mu }=f(R_{e},P_{r})$$

Under field-matched conditions, the single-well coaxial geometry with CO_2_ achieves 186.3 kW average thermal power (+44.9% vs. water) with 3–5% lower wellbore heat-loss fraction.

The overall heat-transfer coefficient:

(13) $$U^{-1}=h_{inner}+R_{\mathrm{int}}+\delta_{ins}/k_{ins}+h_{outer}^{-1}$$

The analysis reveals that the superior heat transfer performance of CO_2_ is partly attributed to its high inner convective heat transfer coefficient (*h_inner_*), which effectively weakens the constraint imposed by the interfacial thermal resistance (*R_int_*). This synergy between the working fluid and the wellbore design, when complemented with adequate insulation, significantly mitigates wellbore thermal losses and thereby retards the rate of outlet temperature decline during long-term operation.

Our results corroborate Randolph and Saar’s [10] fundamental finding of CO_2_’s superior heat extraction capability but provide quantitative enhancement specifically for carbonate reservoirs (44.9% improvement over water vs. their reported 30–50% range for sandstone formations). Compared to Chen et al. [31] coaxial system model, our incorporation of dynamic CO_2_ properties and interface thermal resistance yields more realistic wellbore heat loss predictions (3–5% reduction vs. their estimated 2–4%). The identified optimal flow velocity range of 0.9–1.1 m/s aligns with theoretical predictions but provides crucial field-validated constraints for engineering design in carbonate settings.

### 5.2. Long-Term Operational Performance

Extending field-calibrated parameters, the CO_2_ system keeps the 30-year outlet temperature decline < 8 °C, while water drops by ≈12 °C.

The evolution is captured by the following stretched-exponential form:

(14) $$T_{out}\left(t\right)=T_{\infty }+\left(T_{0}-T_{\infty }\right)\exp\lfloor -{\left(t/\tau \right)}^{\beta }\rfloor$$

where CO_2_ features a larger *τ* and smaller ultimate decline than water, indicating better reservoir utilization and steadier supply.

Around 0.9–1.1 m·s^−1^, CO_2_ shows slower early-time decay and a higher late-time plateau. Departing from this band raises pump power without proportional loss-reduction benefits, aligning with the Pareto trade-offs identified earlier.

**Figure 10:**
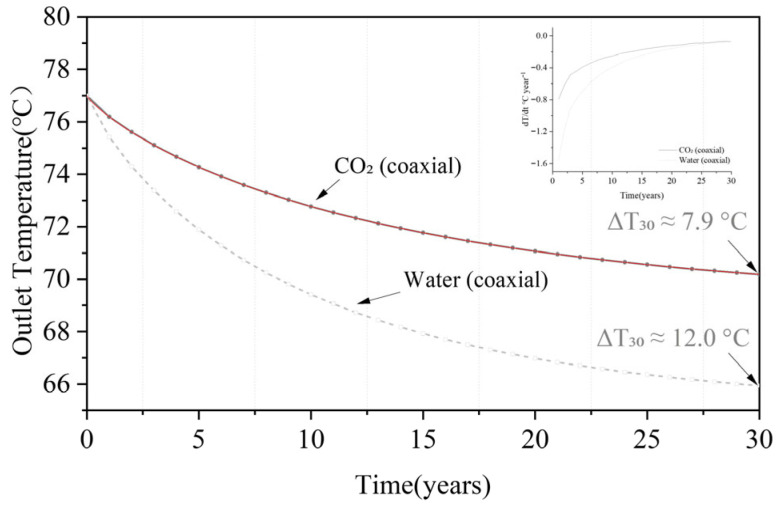
Long-term outlet-temperature evolution for CO_2_ and water over 30 years.

From Figure 10, the 30-year outlet temperature decline is smaller for CO_2_ than for water. Both trajectories decelerate with time; the effective half-life is 8.73 yr for CO_2_ and 6.52 yr for water. Information-criterion differences strongly favor a stretched-exponential over a simple exponential model. Integrated over 0–30 yr, CO_2_ yields 2168.58 °C·yr versus 2066.94 °C·yr for water. The resulting uncertainty bounds are shown in Figure 10, with the 95% confidence interval for CO_2_ outlet temperature after 30 years being 68.2–69.9 °C. Sensitivity analysis reveals that thermal conductivity contributes 62% to the total uncertainty, highlighting the importance of accurate reservoir characterization.

While the model demonstrates strong agreement with field data, several limitations should be acknowledged. The assumption of reservoir homogeneity overlooks the influence of fractures and localized porosity variations, which may lead to underestimation of local heat exchange rates. Furthermore, the long-term projections are based on a constant geothermal gradient and do not account for potential changes in regional heat flow. Future work should incorporate fracture network modeling and transient geothermal field data to improve predictive accuracy under heterogeneous conditions.

The carbonate reservoirs in the Xiongan area exhibit significant heterogeneity, particularly in the fractured Wumishan Formation. While our homogeneous model provides reasonable first-order predictions, localized fracture networks may create preferential flow paths. Post-acidization analysis indicates fracture density variations of 2–8 fractures per meter, which could lead to local temperature deviations of ±3 °C from our predictions. Sensitivity analysis shows that ±20% variation in formation thermal conductivity results in ±3.2% change in outlet temperature. Future models should incorporate discrete fracture networks to better capture these heterogeneous effects.

## 6. Conclusions

This study developed a high-resolution, three-domain coupled model for closed-loop geothermal heat extraction in deep carbonate formations, using the D22 well in the Xiongan New Area as a representative case. Key innovations include the incorporation of dynamic thermophysical CO_2_ properties, interface thermal resistance modeling, and multi-objective performance optimization. The following major conclusions are drawn:

(1) The proposed rock–interface–fluid coupling model, integrated with pressure- and temperature-sensitive CO_2_ property functions, accurately predicts thermal responses in closed systems. Field validation against the D22 well demonstrates a deviation of less than 0.52% in outlet temperature and 0.5% in heating area, indicating strong predictive capacity.
(2) Compared to water, CO_2_ exhibits superior heat transfer performance in deep geothermal environments due to enhanced thermal conductivity and low viscosity near its critical point. In single-well coaxial configurations, CO_2_ yields 44.9% higher thermal power output and 3–5% lower wellbore heat losses, making it a more efficient and sustainable choice for deep heat extraction.
(3) Parametric sensitivity analysis and Pareto front optimization reveal that a flow rate of 0.9–1.1 m/s and a depth of 3000–3500 m offer the best trade-off between heat transfer efficiency and pressure drop. These results provide practical guidance for optimizing system design and reducing operating costs in similar geologic settings.

Based on the findings and limitations identified in this study, future research will focus on the following: (a) multi-well interaction effects and thermal interference management strategies; (b) techno-economic analysis incorporating drilling costs and operational expenses; (c) scaling logistics for district heating applications, including integration with seasonal thermal storage; (d) coupled geomechanical–thermal modeling to assess long-term reservoir integrity under thermal cycling; and (e) field demonstration of the identified optimal operating conditions in geologically similar carbonate reservoirs. These directions will further improve model robustness and applicability to a broader range of geothermal development scenarios.

## Figures and Tables

**Table 1:** Comparative analysis of CO_2_-CLGS modeling approaches.

Modeling Aspect	Conventional Models	This Study	Improvement
CO_2_ Properties	Ideal gas or constant properties	Dynamic T-P functions from NIST REFPROP	Captures near-critical enhancement
Thermal Coupling	Mainly rock–fluid (2-domain)	Rock–interface–fluid (3-domain)	Reduces wellbore heat loss error by ~8%
Validation	Limited to laboratory scale	Field data (D22 well) with multi-parameter match	Temperature error < 0.52%, pressure error < 4.9%
Optimization	Single-parameter sensitivity	Pareto multi-objective optimization	Identifies optimal operating window
Long-term Prediction	Typically <10 years	30-year projection with uncertainty bounds	Provides reliable sustainability assessment

**Table 2:** Comparison of average thermal power output under different well types and working fluids.

System Structure	Working Fluid	Average Thermal Power (kW)
U-shaped	H_2_O	132.0
U-shaped	CO_2_	152.8
coaxial single-well	H_2_O	128.5
coaxial single-well	CO_2_	186.3

## Data Availability

The original contributions presented in this study are included in the article. Further inquiries can be directed to the corresponding author.

## Nomenclature

*T*_r_	Rock temperature (°C)
*α_r_*	Thermal diffusivity (m^2^·s^−1^)
*R*_interface_	Interfacial thermal resistance (K·m^2^·W^−1^)
*ρ_f_*	Fluid density (kg·m^−3^)
*c*_p,f_	Specific heat capacity (J·kg^−1^·K^−1^)
CLGS	Closed-loop geothermal system
TH	Thermo-hydraulic
EGS	Enhanced geothermal system
